# Real-Time and Remote MCMC Trace Inspection with Beastiary

**DOI:** 10.1093/molbev/msac095

**Published:** 2022-05-12

**Authors:** Wytamma Wirth, Sebastian Duchene

**Affiliations:** Peter Doherty Institute for Infection and Immunity, University of Melbourne, Melbourne, Australia; Peter Doherty Institute for Infection and Immunity, University of Melbourne, Melbourne, Australia

**Keywords:** Markov chain Monte Carlo, Bayesian phylogenetics, high performance computing, real-time phylogenetics

## Abstract

Bayesian phylogenetics has gained substantial popularity in the last decade, with most implementations relying on Markov chain Monte Carlo (MCMC). The computational demands of MCMC mean that remote servers are increasingly used. We present Beastiary, a package for real-time and remote inspection of log files generated by MCMC analyses. Beastiary is an easily deployed web-app that can be used to summarize and visualize the output of many popular software packages including BEAST, BEAST2, RevBayes, and MrBayes via a web browser. We describe the design and implementation of Beastiary and some typical use-cases, with a focus on real-time remote monitoring.

## Introduction

Markov chain Monte Carlo (MCMC) algorithms are the driving force behind most modern packages for Bayesian phylogenetics inference (Larget and Simon 1999), although other techniques exist, but have not yet gained the same popularity (e.g., Bouchard-Côté et al. 2012; Fourment et al. 2018; Fourment and Darling 2019). For example, widely used packages, such as BEAST1.10 (Suchard et al. 2018), BEAST2 (Bouckaert et al. 2019), RevBayes (Hohna et al. 2016), and MrBayes (Ronquist et al. 2012), rely on MCMC to sample the posterior distribution. Summarizing and visualizing the posterior samples generated from the MCMC algorithm is central to the interpretation of a Bayesian phylogenetic analysis. Bayesian phylogenetics is increasing in popularity and the way that these analyses are performed is changing. Model complexity and data sets size are increasing. Typically, these large and complex analyses take longer to run and require computational resources that are often only available to research through remote servers (e.g., a high performance computing system).

**Fig. 1.**
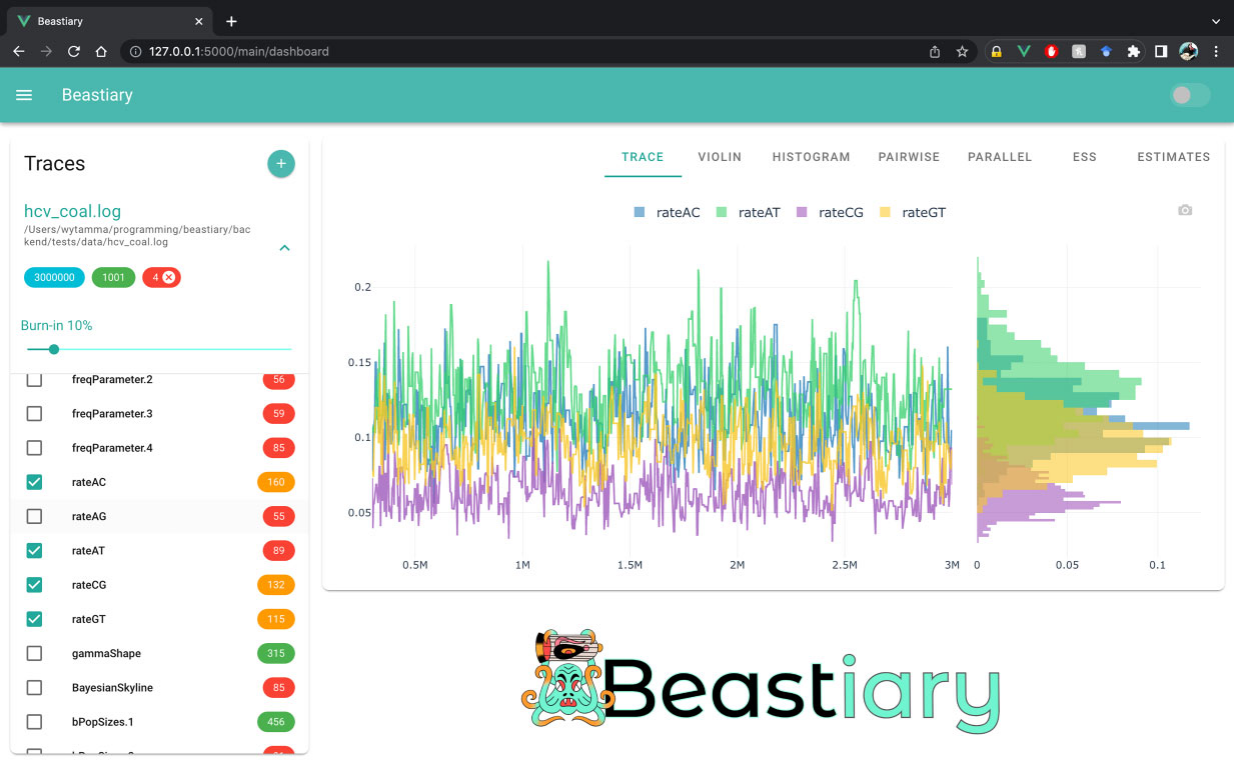
Beastiary front-end main dashboard. The left-hand plane (Traces) shows the number of steps (3,000,000), samples (1,001), and active traces (4) for each log file. Burn-in is set to 10% by default and colour-coded effective sample size (ESS) values are displayed to the right of the trace labels. The right-hand panel show the default trace plot and histograms for each of the selected traces.

While well-established applications for summarizing MCMC outputs exist (Nylander et al. 2008; Warren et al. 2017; Rambaut et al. 2018), these packages lack some features that are becoming more valuable for modern Bayesian phylogenetic analysis [e.g., remote and real-time analysis (Gill et al. 2020)]. To modernize the process of MCMC log file inspection, we have developed Beastiary (version 1.5), a package for real-time and remote interactive data exploration of the output of a Bayesian MCMC analysis (figure 1). Beastiary includes several MCMC diagnostic tools and a focus on functionality for real-time monitoring of analyses on remote servers. Bestiary can read the MCMC log files of BEAST (Drummond and Rambaut 2007), BEAST2 (Bouckaert et al. 2019), RevBayes (Hohna et al. 2016), MrBayes (Ronquist et al. 2012) and any other program that produces white-space delineated log files. Beastiary is easily deployed on remote servers and installed via PYPI with the command pip install beastiary (requires Python version ≥ 3.6.2).

Beastiary is comprised of two parts: the back-end, a web-server that exposes an Application Programming Interface (API) consumed by the front-end, a single page web-app. Beastiary has several features that enhance user experience including dark-mode, exporting plots in SVG format, and exporting summary estimates (e.g., mean, median, and quantiles) in CSV format. Currently bestiary includes trace, violin, histogram, pairwise, parallel coordinate, and cumulative ESS plots, with several others expected to be added in future updates (see documentation https://beastiary.wytamma.com).

A typical use case for beastiary would involve starting an analysis by submitting it to a high performance computer (HPC) queue. When running an analysis on a HPC one would normally wait until the analysis has finished before inspecting the output or download the partial log file before the analysis finishes. However, with beastiary one can inspect an MCMC analysis and determine if it has converged (or not) in real-time. A researcher could run beastiary *.log to tell beastiary to watch all the “.log” files in the current directory (see documentation for detailed commands). The researcher then navigates to local-host port 5000, that is, http://127.0.0.1:5000, and inspects their analysis using the beastiary web-app (see documentation for port forwarding example). The web-app can be used to confirm that multiple independent runs have converged to the same distribution and all parameters have ESS values of at least 200. A screen capture of the remote and real-time utility of beastiary can be found at https://youtu.be/y6i_UCCQTso (or in the supplementary video S1, Supplementary Material online).

Because Beastiary is essentially a web-server it can be deployed to many different computing environments, leading to some interesting use-cases. For example, beastiary can be run in Google Colab notebooks. We have provided a notebook to run BEAST in a cloud computing environment (currently free of charge). This notebook takes advantage of the GPUs provided by Google and uses beastiary to visualize the results in real-time and can be found at https://colab.research.google.{PI}com/gist/Wytamma/67bdaa46f7c3c64616592e6a8fc23f4d/beastiary.ipynb (or in the Supplementary material online).

The real-time MCMC inspection utility of beastiary can be extremely valuable for determining when an MCMC analysis should be stopped. Many analyses are run on HPCs and so the remote feature of beastiary enables users to analyse output without having to copy them to their personal computer (e.g., for use with Tracer). Beastiary is not designed to replace currently available software. For example, Tracer has functions to visualize Bayesian skyline plots and model-fit statistics (Drummond et al. 2005; Rambaut et al. 2018), while RWTY has useful tools to assess the effective sample size of tree topologies (Lanfear et al. 2016; Warren et al. 2017). Instead, the purpose of Beastiary is to fill the need of real-time and remote trace inspection, which we expect to grow with the increasing use of remote servers for phylogenetic analyses.

Beastiary source code is freely available via GitHub at: https://github.com/Wytamma/beastiary. Extensive beastiary documentation can be found at: https://beastiary.wytamma.com.

## Supplementary Material

msac095_Supplementary_Data

## Data Availability

Beastiary source code is freely available via GitHub at: https://github.com/Wytamma/beastiary. Extensive beastiary documentation can be found at: https://beastiary.wytamma.com.
